# Towns with extremely low mortality due to ischemic heart disease in Spain

**DOI:** 10.1186/1471-2458-12-174

**Published:** 2012-03-09

**Authors:** María José Medrano, Raquel Boix, Alba Palmera, Rebeca Ramis, Iñaki Galán, Gonzalo López-Abente

**Affiliations:** 1 Centro Nacional de Epidemiología, Instituto de Salud Carlos III, Madrid, Spain

**Keywords:** Ischemic heart disease, Mortality, Spatial analysis, Spain

## Abstract

**Background:**

The cause of coronary disease inframortality in Spain is unknown. The aim of this study is to identify Spanish towns with very low ischemic heart disease mortality, describe their health and social characteristics, and analyze the relationship with a series of contextual factors.

**Methods:**

We obtained the number of deaths registered for each of 8,122 Spanish towns in the periods 1989-1998 and 1999-2003. Expected deaths, standardized mortality ratio (SMR), smoothed Relative Risk (RR), and Posterior Probability (PP) of RR > 1 were calculated using Bayesian hierarchical models. Inframortality was defined as any town that displayed an RR below the 10^th ^percentile, an SMR of under 1 for both sexes, and a PP of RR > 1 less than or equal to 0.002 for male and 0.005 for female mortality, during the two periods covered. All the remaining towns, except for those with high mortality classified as "tourist towns", were selected as controls. The association among socioeconomic, health, dietary, lifestyle and vascular risk factors was analyzed using sequential mixed logistic regression models, with province as the random-effects variable.

**Results:**

We identified 32 towns in which ischemic heart disease mortality was half the national rate and four times lower than the European Union rate, situated in lightly populated provinces spread across the northern half of Spain, and revealed a surprising pattern of geographic aggegation for 23 of the 32 towns. Variables related with inframortality were: a less aged population (OR 0.93, 95% CI 0.89-0.99); a contextual dietary pattern marked by a high fish content (OR 2.13, 95% CI 1.38-3.28) and wine consumption (OR 1.50, 95% CI 1.08-2.07); and a low prevalence of obesity (OR 0.47, 95% CI 0.22-1.01); and, in the case of towns of over 1000 inhabitants, a higher physician-population ratio (OR 3.80, 95% CI 1.17-12.3).

**Conclusions:**

Results indicate that dietary and health care factors have an influence on inframortality. The geographical aggregation suggests that other factors with a spatial pattern, e.g., genetic or environmental might also be implicated. These results will have to be confirmed by studies *in situ*, with objective measurements at an individual level.

## Background

Ischemic heart disease mortality in Spain is very low in comparison with other countries in the European region [1,2]. Domestic distribution is not uniform, however, so that provinces with lower mortality register rates that are 40% lower than the national rate [3]. Spanish mortality studies at a municipal level show that coronary mortality displays very wide variability, even after the use of smoothing techniques [4,5]. Hence, there are large areas of coronary inframortality in Spain, and within these, there are towns whose populations show an extremely low risk of dying from coronary disease.

To our knowledge, no study has previously sought to characterize inframortality due to ischemic heart disease in Spain. Studies that have analyzed the geographical distribution of vascular mortality and other causes of death, have focused on identifying areas with excess mortality [6,7] or been targeted at studying the impact of specific factors, such as social inequalities [8-10] and environmental [11-14] or dietary factors, [15-17] on increased risk of dying. At an international level, what are now regarded as classic ecological studies [18,19] drew attention to the lower mortality in southern European countries, but analyses on the Mediterranean paradox and its link to diet and hypercholesterolemia [20-22] used population clusters that were too large and, by extension, too heterogeneous (e.g. country), and failed to take multifactorial and contextual aspects of the disease into account. Many studies have shown that the socioeconomic characteristics of the local setting have an influence on individual cardiovascular risk, even when adjustment is made for individual risk [23].

In the last decade, the development of spatial epidemiology and epidemiology of the social context has generated new theoretical and methodological frameworks, which enable the study of low coronary mortality to be addressed with a new approach of greater potential [24].

This study sought to identify Spanish towns with very low ischemic heart disease mortality, describe their health care and social characteristics, and analyze the relationship with different contextual factors.

## Methods

### Mortality

This study was based on a small-area spatial analysis of mortality, with the study units being Spanin's 8,073 towns as per the 1991 and 2001 Population Censuses [25].

The study analyzed mortality due to ischemic heart disease (codes 410-414 of the International Classification of Diseases 9^th ^Revision (ICD-9) and codes I20-I25 of the ICD-10) corresponding to two periods, 1989-1998 and 1999-2003. Deaths registered at a municipal level, broken down by sex and age, were obtained from individual anonymized files furnished by the National Statistics Institute (*Instituto Nacional de Estadística*-*INE*). Age and sex population data in each of the two periods (1989-1998 and 1999-2003), were drawn from the 1991 and 2001 census respectively, so changes in population figures and age structure were taken into account in mortality calculations.

The following were calculated for each town, for each of the two periods studied, and for men and women separately: a) expected deaths, using the overall national age- and sex-specific rates as reference; b) standardized mortality ratio (SMR); and c) smoothed Relative Risk (RR) and Posterior Probability (PP) of RR > 1, using the Besag, York and Mollié hierarchical model fitted using Markov chain Monte Carlo simulation methods [26]. The spatial distribution of the RRs was plotted on maps using geographic information systems.

### Definition of inframortality

The criteria used to define ischemic heart disease inframortality were: a) a smoothed Relative Risk below the 10^th ^percentile of the distribution of municipal coronary mortality nationwide; b) an SMR of less than 1 in both sexes; and c) a Posterior Probability of RR > 1 less than or equal to 0.002 for male and 0.005 for female mortality (due to the lower number of deaths registered). Towns that met these three criteria in the two target periods were identified and then selected separately for each sex to ensure representation of female inframortality. A total of 32 towns were selected.

The remaining towns in Spain (n 7,953) were taken as controls, with high-mortality towns classified as "tourist towns" by the National Statistics Institute in the 2008 Hotel Occupation Survey [27] (88 towns) being excluded to avoid possible errors of classification of the variable "residence", and/or imported mortality [28].

### Characteristics studied

#### a) Socioeconomic indicators at a municipal level

Number of inhabitants, percentage of the population over the age of 65 years, percentage of illiteracy, percentage of unemployment, percentage of the population engaged in farming, and number of inhabitants per province, based in all cases on data provided by the Census [25] (data refer to 2001).

Rurality index [29]. This is a complex indicator that takes the following into account: population density; population aged over 65 years and under 14 years; dependency index; retirement index; population devoted to the primary sector; and habitability of dwellings. It has a rising scale, ranging from -3.50 (minimum rurality) to 3.78 (maximum rurality), with a mean of 0 (data refer to 2001).

Deprivation index [30]. This takes into account the percentages of unemployment, illiteracy and manual workers. It ranges from -3.76 (minimum deprivation) to 5.06 (maximum deprivation), with a mean of 0 (data refer to 2001).

Socioeconomic level [31]. This is drawn up on the basis of occupation and professional status (data refer to 2001).

Level of disposable family income per inhabitant, [32] an estimate based on provincial disposable family income and the following municipal data: telephones installed in private homes; broadband internet; proportion of inhabitants with higher education; proportion of skilled workers; proportion of job seekers; distance to main town in the area; and mean cost of housing. This indicator, available solely for towns of over 1,000 inhabitants, was imputed for towns with smaller populations (n 4,909, total population 1,541,626 inhabitants) using linear regression and the National Statistics Institute's indicator of socioeconomic status (r 0.89) (data refer to 2003).

Industrial index, [32] i.e., town revenue deriving from business tax (*Impuesto de Actividades Económicas*) levied on local industrial activities, divided by the total business-tax revenue for the country as a whole. This indicates the town's relative ranking in national industry and was only available for towns of over 1,000 inhabitants (data refer to 2003).

#### b) Health-care indicators

Number of licensed physicians in the province per 1,000 population, [33] hospital beds in the province per 1,000 population, and high-tech resources (hemodynamics and angiography units) per million population [34] (data refer to 2001).

#### c) Dietary factors

Composition of the provincial family diet per person per day, [35] based on food purchased for home consumption, in terms of total calories (kilocalories), total fat, fish, folates, olive oil, wine and alcohol (grams)(data refer to 1991).

#### d) Mortality pattern, longevity, competitive mortality, and quality of certification of cause of death

SMR of all-cause municipal mortality; mean age at death; mortality due to malignant tumors; and mortality due to ill-defined causes. Deaths registered at a municipal level, broken down by sex and age, were obtained from individual anonymized files furnished by the National Statistics Institute (data refer to 2001).

#### e) Cardiovascular risk factors

Provincial age- and sex-adjusted prevalence of arterial hypertension, hypercholesterolemia, obesity, diabetes, leisure-time sedentarism and smoking among the population aged over 20 years [36] (self-reported data refer to 1999).

### Statistical analysis

Variance analysis was used to compare low-mortality towns to the remaining towns by reference to the factors considered. To control for any potential confounding biases stemming from associations among the factors analyzed, mixed-effects logistic regression models were fitted, taking coronary mortality as the dichotomous dependent variable (low-mortality towns versus remaining towns), with province introduced as a random-effects term. The following three sequential logistic models were constructed: the first was adjusted for demographic and socioeconomic factors, taking the socioeconomic index (estimated disposable family income), rather than individual variables, as a complex indicator, in order to obtain models that were more parsimonious; the second was additionally adjusted for dietary variables and prevalence of cardiovascular risk factors estimated at a provincial level; and lastly, the third model was, in turn, additionally adjusted for prevalence of protective dietary factors (consumption of wine and fish) and health-care resources. To prevent overadjustment for redundant data in the case of the variables "total calories" and "wine consumption", the variables "total fat" and "alcohol consumption" were excluded.

### Sensitivity analyses

In view of the fact that mortality rates in small populations can be unstable, even when multi-annual periods are pooled, the analysis was repeated, by restricting it to towns of over 1,000 inhabitants.

In the same manner, the restrictive criteria used to define inframortality make that towns in which there are very few inhabitants and no coronary deaths are not selected because they fail to meet the significance criterion (PP of RR > 1), so the analysis was also repeated including the 397 towns with zero deaths as inframortality towns, irrespective of their PP of RR > 1 value.

The study was approved by the Research Committee of the Carlos III Institute of Health and complies with the statutory criteria for statistical secrecy, in accordance with the National Statistics Institute's data-release protocol.

## Results

Table 1 shows the population data, location, and ischemic heart disease mortality of the 32 towns selected. The overall number of deaths due to ischemic heart disease in these 32 towns was 443 versus the figure of 850 which would have been expected, had their age-specific mortality been equal to that of Spain as a whole. In 2001, the mortality rates (mean ± standard deviation, rates per 100,000 population, adjusted to the standard European population) were 43.4 ± 20.6 in men and 16.3 ± 9.6 in women, notably lower than national rates (91.6 and 39.5, respectively) and those of the 27 European Union countries (153.9 and 78.4, respectively).

**Table 1:** Towns with extremely low mortality due to ischemic heart disease

ARs/TOWN	PROVINCE	POPULATION	MORTALITY 1999-2003							
			MEN					WOMEN		
			RATE*	SMR	RR	PP	RATE	SMR	RR	PP
**Castile-León**										
Aranda de Duero	Burgos	30,875	63.7	0.70	0.68	0.0000	23.4	0.57	0.57	0.0002
Salas de los Infantes	Burgos	2,085	18.7	0.21	0.56	0.0018	19.7	0.48	0.58	0.0106
Estepar	Burgos	797	20.8	0.23	0.62	0.0008	0.0	0.00	0.53	0.0018
Balbases (Los)	Burgos	341	0.0	0.00	0.63	0.0036	34.3	0.84	0.57	0.0050
Castrojeriz	Burgos	971	70.8	0.78	0.69	0.0038	8.3	0.22	0.53	0.0006
Pampliega	Burgos	394	0.0	0.00	0.61	0.0034	0.0	0.00	0.53	0.0042
Santa Maria del Campo	Burgos	710	66.7	0.74	0.65	0.0038	31.9	0.78	0.57	0.0042
Villadiego	Burgos	1,924	51.5	0.46	0.66	0.0032	48.8	0.60	0.57	0.0038
Villahoz	Burgos	402	69.0	0.76	0.65	0.0090	0.0	0.00	0.54	0.0040
Merindad de Río Ubierna	Burgos	1,330	45.5	0.50	0.66	0.0026	9.4	0.23	0.58	0.0038
Aguilar de Campoo	Palencia	7,435	55.1	0.61	0.70	0.0026	17.1	0.42	0.52	0.0000
Alar del Rey	Palencia	1,223	40.4	0.45	0.71	0.0200	13.1	0.32	0.54	0.0018
Cervera de Pisuerga	Palencia	2,586	60.0	0.66	0.77	0.0334	17.5	0.43	0.58	0.0022
Aldeadavila de la Ribera	Salamanca	1,540	63.5	0.70	0.65	0.0150	12.8	0.31	0.50	0.0034
San Pedro de Gaillos	Segovia	337	55.8	0.62	0.67	0.0088	0.0	0.00	0.56	0.0042
Berlanga de Duero	Soria	1,100	61.5	0.68	0.75	0.0296	20.3	0.49	0.60	0.0048
**Castile-La Mancha**										
Palomares del Campo	Cuenca	928	0.0	0.00	0.60	0.0012	11.6	0.28	0.60	0.0100
Torrejoncillo del Rey	Cuenca	652	30.2	0.33	0.61	0.0008	25.7	0.63	0.62	0.0128
Sotorribas	Cuenca	955	12.6	0.14	0.62	0.0020	9.6	0.23	0.56	0.0030
Quintanar del Rey	Cuenca	7,254	50.3	0.56	0.65	0.0016	35.2	0.86	0.79	0.0992
**Aragon**										
Albarracín	Teruel	1,025	62.9	0.69	0.65	0.0004	20.9	0.51	0.64	0.0096
Monreal del Campo	Teruel	2,391	30.8	0.34	0.61	0.0012	21.7	0.53	0.61	0.0102
**Galicia**										
Barco de Valdeorras	Orense	13,518	51.7	0.57	0.65	0.0002	29.0	0.71	0.70	0.0180
Saviñao (O)	Lugo	5,010	42.5	0.47	0.66	0.0010	18.5	0.45	0.58	0.0004
Covelo (O)	Pontevedra	3,743	44.1	0.49	0.76	0.0332	18.6	0.45	0.62	0.0024
Lalin	Pontevedra	19,869	58.0	0.63	0.70	0.0004	21.0	0.51	0.62	0.0000
**Catalonia**										
Deltebre	Tarragona	10,757	38.9	0.43	0.60	0.0000	15.2	0.37	0.60	0.0028
**Cantabria**										
Campoo de Enmedio	Cantabria	3,928	46.0	0.51	0.65	0.0018	13.1	0.32	0.54	0.0006
Suances	Cantabria	6,573	65.4	0.72	0.73	0.0320	21.2	0.52	0.56	0.0024
Valderredible	Cantabria	1,120	46.03	0.51	0.68	0.0050	12.5	0.31	0.54	0.0014
**Navarre**										
Puente La Reina	Navarre	2,412	28.4	0.31	0.81	0.0898	7.6	0.19	0.58	0.0050
Yerri	Navarre	1,543	48.2	0.53	0.81	0.0898	7.6	0.19	0.56	0.0040

* Rate adjusted according to the standard European population

ARs: Autonomous Regions

SMR: Standardized Mortality Ratio

RR: Relative Risk

PP: Posterior Probability of RR > 1

These towns were distributed across the northern half of the country, and belonged to the provinces of Burgos (10), Cuenca (5), Cantabria (3), Palencia (3), Teruel (2), Navarre (2), Pontevedra (2), Lugo (1), Orense (1), Tarragona (1), Salamanca (1), Segovia (1) and Soria (1). Only two were situated on the coast (Suances in Cantabria, and Deltebre in Tarragona).

**Figure 1:**
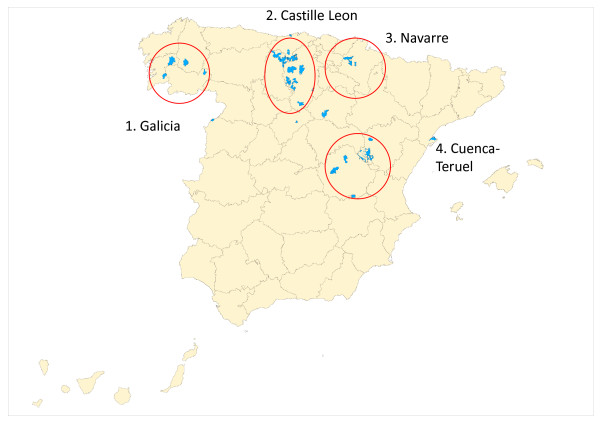
**Geographic distribution of towns with extremely low mortality due to ischemic heart disease in Spain**.

The map plotted in Figure 1 shows that 23 of the 32 towns with inframortality were geographically grouped into four areas, namely: central Galicia (*marked in the figure as area 1*); provinces of Burgos and Palencia in northern Castille-Leon (area 2); province of Cuenca (area 3); and western Navarre (area 4).

In comparison with the remainder (Table 2), towns with coronary inframortality were located in provinces with low population density (p 0.023), and had few inhabitants, though the difference vis-à-vis the mean size of the country's remaining towns did not prove statistically significant. Insofar as socioeconomic characteristics were concerned, the towns selected displayed lower proportions of illiteracy, unemployment, aged population, population engaged in farming, and deprivation index, and a higher socioeconomic index of disposable family income. These differences were small, however, and failed to reach statistical significance, with the exception of the lower proportion of the economically active population devoted to farming (28.5% versus 36.0%, p 0.046), and with marginal statistical significance, the smaller proportion of unemployed population (11.6% versus 15.4, p 0.066).

**Table 2:** Characteristics in towns with coronary inframortality compared with the country's remaining towns

	TOWNS WITH CORONARY INFRAMORTALITY		REMAINING TOWNS		
	(n = 32)		(n = 7953)		
	Mean	SD	Mean	SD	p
Population	4,164	6,357	4,541	42,266	0.960
Provincial population	359,351	200,638	826,639	1,166,109	0.023
Socioeconomic indicators *					
% Illiteracy	2.7	3.6	3.3	3.8	0.307
% Unemployment	11.6	4.3	15.4	11.7	0.066
% Agriculture	28.5	16.9	36.0	21.4	0.046
% Population > = 65 years	22.7	7.3	23.5	9.5	0.635
Rurality index	-0.1	0.8	0.0	1.0	0.486
Deprivation index	-0.2	0.5	0.0	1.0	0.174
Socioeconomic index	1.0	0.1	1.0	0.2	0.173
Index of disposable family	5.3	1.4	4.8	2.0	0.156
income					
**Health indicators**					
Licensed physicians per province/1000	4.6	0.8	4.6	0.9	0.921
Hospital beds per province/1000	4.2	0.9	4.0	0.8	0.101
Hemodynamic units/10^6^	0.9	1.5	2.3	2.2	0.001
Angiography units/10^6^	2.3	2.1	2.6	2.5	0.450
**Content of diet (gr/person/day)**					
Total calories, Kcal/per/day	2,716.0	275.9	2,626.5	234.7	0.032
Total fat	34.5	6.9	33.0	7.6	0.251
Fish	93.6	21.7	75.7	14.7	< 0.001
Folic acid	2.8	1.6	3.3	1.4	0.060
Olive oil	34.5	6.9	33.0	7.6	0.252
Wine	101.1	57.8	72.2	40.5	< 0.001
Alcohol	12.4	6.2	9.7	4.3	< 0.001
Prevalence of vascular risk factors** (%)					
Arterial hypertension	20.7	2.4	20.6	2.5	0.809
Hypercholesterolemia	9.1	1.3	9.4	1.6	0.216
Obesity	10.7	1.7	11.7	2.2	0.007
Diabetes mellitus	6.4	1.1	7.3	1.7	0.002
Sedentarism	39.6	7.6	42.5	8.7	0.059
Smoking	27.2	2.4	28.1	2.3	0.040
**Pattern of mortality and competitive mortality (SMR)**					
General mortality men	0.8	0.3	0.8	0.8	0.638
General mortality women	0.8	0.3	0.9	0.8	0.587
Age at death	77.6	15.3	75.7	15.8	< 0.001
Tumor-related mortality	0.8	0.2	0.8	0.6	0.782
Ill-defined causes of death	0.3	0.2	0.3	0.4	0.692

* Complex socioeconomic indices: see description in Materials and Methods section

**Adjusted for age and sex. Self-reported data.

SD: Standard deviation.

SMR: Standardized Mortality Ratio.

The dietary pattern of the geographic setting of these towns was characterized by a calorie content that was slightly higher than the mean. Fish and wine consumption was much higher in towns with inframortality, 23.6% and 40% respectively (p < 0.001), as was that of alcohol in general (27.8% p < 0.001). Although the prevalences of smoking, sedentarism, obesity and diabetes were significantly lower than those of controls from a statistical standpoint, the difference was small (1%). No differences were observed in the prevalence of perceived arterial hypertension and hypercholesterolemia.

All-cause and tumor-related mortality were 20% lower than the national standard and equal to the mean SMR of the remaining towns. Longevity was longer by 2 years in such towns. Furthermore, the physician-population ratio was equal to and the hospital bed-population ratio was higher than that of control towns, and mortality due to ill-defined causes was 70% lower than the national standard.

Of the factors considered, those that went furthest to explain municipal inframortality (Table 3) were, at a local level, the lower proportion of the population aged 65 years and over, and at a provincial level, the lower prevalence of obesity and the higher consumption of wine and, in particular, fish. No significant associations were found with the frequencies of arterial hypertension, hypercholesterolemia or smoking. Paradoxically, frequency of leisure-time sedentarism was positively associated with coronary inframortality. The fitting sequence showed that the absence of association of some risk factors in model 2 was caused by confounding due to the effect of the protective factors.

**Table 3:** Factors associated with extremely low coronary mortality.

		Model 1			Model 2			Model 3	
	OR	(95% CI)	P	OR	(95% CI)	p	OR	(95% CI)	p
Social factors									
Provincial population (x10,000)	0.97	(0.95-0.99)	0.035	0.98	(0.96-1.01)	0.204	0.97	(0.94-1.00)	0.058
Index of disposable family income*	1.09	(0.85-1.40)	0.486	1.04	(0.80-1.34)	0.790	0.96	(0.73-1.27)	0.789
Population > = 65 years (%)	0.94	(0.90-0.99)	0.023	0.94	(0.89-0.98)	0.013	0.93	(0.89-0.99)	0.012
Lifestyle and vascular risk factors									
Sedentarism (%)	-		-	1.07	(0.94-1.22)	0.289	1.21	(1.01-1.45)	0.036
Calorie content diet (x 100 Kcal/inhabitant/day)	-		-	1.23	(0.92-1.65)	0.158	0.59	(0.33-1.03)	0.063
Obesity (%)	-		-	0.70	(0.40-1.21)	0.204	0.47	(0.22-1.01)	0.053
Arterial hypertension (%)	-		-	1.08	(0.84-1.41)	0.529	1.23	(0.97-1.56)	0.093
Hypercholesterolemia (%)	-		-	0.81	(0.53-1.22)	0.313	0.97	(0.62-1.52)	0.905
Diabetes mellitus (%)	-		-	0.66	(0.39-1.10)	0.113	0.83	(0.47-1.46)	0.527
Smoking (%)	-		-	0.97	(0.72-1.32)	0.857	1.18	(0.90-1.55)	0.235
Protective factors									
Wine content diet (x 10 gr/inhabitant/day)	-		-	-		-	1.50	(1.08-2.07)	0.015
Fish content diet(x10 gr/inhabitant/day)	-		-	-		-	2.13	(1.38-3.28)	0.001
Folate content diet							1.20	(0.73-2.00)	0.471
Physicians per province (x1,000 inhabitants)	-		-	-		-	2.14	(0.74-6.16)	0.160
Hospital beds per province (x1,000 inhabitants)	-		-	-		-	0.59	(0.24-1.46)	0.253

Multivariate analysis (n 7985)

OR: Odds Ratio, derived from mixed-effects logistic regression models, adjusted for:

Model 1: demographic and socioeconomic factors.

Model 2: factors present in model 1 + diet and prevalence of cardiovascular risk factors.

Model 3: factors present in model 2 + protective factors in the diet and health care resources.

*Index of disposable family income: see description in Methods section

The results of the two sensitivity analyses conducted are presented in Table 4. First, the analysis was repeated, solely considering towns of over 1000 inhabitants (Analysis I) and adding indicators, such as the industrial index, which were exclusively available for this group of towns. The results were similar but the provincial physician-population ratio was strongly associated with municipal inframortality. Second, we conducted a sensitivity analysis modifying the selection criteria to test if selection of these towns affect the results (Analysis II). Although this new analysis was based in a quite different set of towns with different characteristics (ie, small villages with older population and higher income), the main results, namely diet as the factor most associated with low mortality, were basically unchanged.

**Table 4:** Sensitivity analyses.

		Sensitivity analysis I			Sensitivity analysis II	
	OR	(95% CI)	p	OR	(95% CI)	p
Social factors						
Provincial population (x10,000)	0.95	(0.92-0.98)	0.005	0.99	(0.99-1.00)	0.006
Index of disposable family income*	1.18	(0.75-1.86)	0.463	1.19	(1.11-1.28)	< 0.001
Population > = 65 years (%)	1.06	(0.97-1.15)	0.166	1.03	(1.02-1.04)	< 0.001
Industrial Index**	1.00	0.34-3.41	0.670	-	-	-
Lifestyle and vascular risk factors						
Sedentarism (%)	1.15	(0.95-1.38)	0.150	1.00	(0.96-1.05)	0.700
Calorie content diet (x 100 Kcal/inhabitant/day)	0.60	(0.32-1.11)	0.108	0.82	(0.72-0.95)	0.008
Obesity (%)	0.64	(0.27-1.49)	0.303	0.70	(0.57-0.85)	< 0.001
Arterial hypertension (%)	1.12	(0.81-1.53)	0.491	1.08	(0.98-1.18)	0.113
Hypercholesterolemia (%)	1.08	(0.64-1.80)	0.782	1.03	(0.90-1.19)	0.638
Diabetes mellitus (%)	1.19	(0.65-2.19)	0.567	0.87	(0.73-1.04)	0.140
Smoking (%)	1.06	(0.78-1.45)	0.702	1.11	(0.90-1.55)	0.235
Protective factors						
Wine content diet (x 10 gr/inhabitant/day)	1.31	(0.93-1.84)	0.120	1.02	(0.94-1.12)	0.531
Fish content diet(x10 gr/inhabitant/day)	2.30	(1.44-3.68)	< 0.001	1.39	(1.16-1.66)	< 0.001
Folate content diet	0.98	(0.54-1.76)	0.940	1.35	(1.15-1.60)	< 0.001
Physicians per province (x1,000 inhabitants)	3.80	(1.17-12.3)	0.026	0.90	(0.65-1.25)	0.546
Hospital beds per province (x1,000 inhabitants)	1.08	(0.34-3.41)	0.894	0.87	(0.65-1.15)	0.346

Factors associated with extremely low coronary mortality. Analysis I: multivariate analysis confined to towns of over 1,000 inhabitants (n 3,074). Analysis II: multivariate analysis with alternative definition criteria, including towns with zero deaths (n 397) as inframortality towns

OR: Odds Ratio, derived from mixed-effects logistic regression models, adjusted for variables in the table.

* Index of disposable family income: see description in Methods section

** Industrial index only available for towns over 1,000 inhabitants. See description in Methods section

## Discussion

The cause of inframortality due to ischemic heart disease in southern European countries - arguably their most relevant epidemiological characteristic- is unknown. Thanks to currently available spatial techniques of analysis, this study was able to identify populations that displayed this characteristic in the extreme and maintained it over a period of 15 years. Such identification is of multifold interest, inasmuch as it enables scientific hypotheses to be generated, pinpoints a proposed site for future studies, and corroborates the relationship between ischemic heart disease mortality and the characteristics of the social context.

The results suggest that coronary inframortality in the towns selected, half the national rate and as much as 5 times lower than the rate of northern European countries, [1] is not attributable to underdiagnosis resulting from lack of health care resources, poor quality in the certification of cause of death, or competitive mortality because mortality due to other causes, and to cancer in particular, is likewise low. The greater longevity of the inhabitants of these towns serves to further support the fact that coronary inframortality is real, since there is practically no possibility of there being an unregistered death in Spain. It also implies that these populations exhibit a low risk of dying for any cause, fact that may be explained by many vascular risk and protective factors being common to other diseases, in fact the most frequent ones, such as cerebrovascular disease and cancer

Geographically, these towns with extremely low ischemic heart disease mortality are situated in sparsely populated provinces in the northern half of the country and, unexpectedly, reveal a trend towards geographical aggregation. Although the towns identified are located within the large areas of low mortality shown on official atlases [4,5] and the methods used favor the appearance of geographical aggegations, the possibility cannot be ruled out that grouping may be due to the presence of factors with a spatial pattern -whether of a protective nature or entailing lower exposure to risk factors- which were not taken into account in the analysis. Hence, certain environmental factors (such as climatic factors, composition of the local drinking-water supply or environmental pollution) could not be considered, since these data were unavailable for many towns in the country, including many of the towns identified. Similarly, genetic factors could not be considered and, while genetic population studies undertaken in Spain show that the Spanish population is homogeneous in terms of overall genetic structure, fine structure analyses nevertheless reveal a geographic variation that may be more evident in small, rural or isolated samples [37]. Consequently, one cannot rule out the possibility that, in the case grouping of small towns, there may be some genetic characteristic which confers low vascular risk. One example of a town having low mortality attributable to genetic features is Limone sul Garda, a small town in the north of Italy, whose inhabitants present with a mutation in the apolipoprotein A1 (ApoA1), which confers cardiovascular protection and increases longevity [38,39]. Taking all together, the results indicate that further in-depth studies of this spatial agregation are needed.

With respect to the characterization of these towns, when compared to the rest of the country, inframortality was linked to their having: a less aged population structure; a contextual dietary pattern characterized by a higher fish and wine content, lower calorie content and a lower prevalence of obesity; and, in towns of over 1,000 inhabitants, a higher physician-population ratio. In line with the results of earlier studies on social inequalities and health in Spain, [40-42] the strength of association between economic level and coronary inframortality decreased when adjustment was made for diet and vascular risk factors, a finding which might be interpreted as indicating that the latter are intermediate factors and a possible explanation for this association [43]. Nevertheless, the difference in income between these and the remaining towns is small: in other words, they are towns with an income level which, albeit higher, is not excessively so.

Low prevalence of obesity and high consumption of fish appear as the variables most closely associated with coronary inframortality. The effect of both of these on risk of coronary disease has been clearly demonstrated in prospective population studies conducted in a number of countries [44,45] and is also reflected at a population-cluster level in ecologic studies [46]. In Spain, fish consumption is very high in comparison with other western countries, with the communities of Cantabria and Castile-León registering the highest intake [35]. The high mortality of some Spanish provinces has already been associated with lower fish consumption by previous studies [17]. Insofar as obesity is concerned, the result is coherent with recent studies, which reckon that, in the Spanish population, risk of ischemic heart disease attributable to excessive weight can be assumed to be very high and even higher than that posed by the classic risk factors [47,48]. While mean calorie intake per person per day was somewhat higher (90 Kcal) in towns with inframortality than in the remaining towns, this difference might nevertheless be accounted for by a higher wine consumption. Indeed, the adjusted results show an inverse association between calorie intake and inframortality. Consumption of wine also showed an association with coronary inframortality, in agreement with many studies that have reported the protective effect of moderate wine consumption [17,49,50]. The positive association between leisure-time sedentarism and inframortality is paradoxical and can only be interpreted by uncontrolled confounding, e.g., due to physical exercise during the work day.

The explanation for the low coronary mortality of these towns does not lie in differences in the prevalence of arterial hypertension and hypercholesterolemia or diabetes. This may be real or, alternatively, it may be an artifact, due to the data having been drawn from a survey [36] and the fact that the prevalence reported is perceived, i.e., diagnosed and possibly treated. Similarly, these towns' low coronary mortality is not explained by differences in the prevalence of smoking.

Lastly, not only are the health care resources of towns with coronary inframortality no greater than those of the rest of the towns, but they actually have fewer high-tech resources, since such resources have been placed in high mortality areas. Nevertheless, when adjustment was made for the remaining factors and the analysis was confined to towns with more stable mortality figures, the physician-population ratio was observed to be positively associated with inframortality in towns with over 1,000 inhabitants.

The possibility of results being biased because the restrictive criteria used to define inframortality can be ruled out as sensitivity analysis modifying the selection criteria to include 397 towns in which there were no coronary death for 15 years yieded similar results, namely dietary factors as the factor most associated with low mortality.

The variables analyzed were based on the information available, in some cases incomplete, such as environmental data, or nonexistent, such as genetic data. While obtaining these data for all Spanish towns is not feasible, studying representative population samples of such towns and of other control towns is possible. This study enables future studies to be steered in this direction. Similarly, there is no data at a municipal level on the factors most directly implicated, such as diet or the prevalence of vascular risk factors. In such cases, provincial data were used by way of giving a description of the context in the absence of the pertinent data. The use of multilevel and spatial models minimizes the biases of this approach [51]. Furthermore, in aspects such as diet and its health-related consequences, the context not only determines individual behavior, but also has an influence on cardiovascular risk, even when individual risk is adjusted for [23,24]. However, although we used hierarchical regression models with province-specific random intercepts to adjust the association between municipal coronary mortality for between-province differences in any relevant factor, residual confounding induced by within-province variations in cardiovascular risk factors and diet cannot be ruled out. Lastly, the dietary variables, coming from the only nation-wide nutritional study available in Spain, refer to 1991 which is eight years before the beginning of the study period, and can be temporarily inadequate to mortality data. Nevertheless, dietary factors take time to cause coronary disease and to lead to death, so an eight years lag can be regarded as adequate. Moreover, the nutrition data used are not intake measures, but the composition of the dietary pattern in households, which do not change in short time. Despite these considerations, the stength of the dietary associations with low coronary mortality found in this study deserves further investigations with in depth nutritional study.

The fact of that this was an ecologic study is somewhat irrelevant insofar as the aspects relating to causality are concerned, given that in all the factors considered the causal association is clearly established. However, analysis with aggregate data, coupled with the nature of the data used and the lack of possibly relevant information, render it difficult for statistical significance to be achieved in the explanatory analyses.

## Conclusions

In brief, towns with extremely low coronary mortality were identified. While the results show that dietary factors are linked to inframortality, the geographical aggregation suggests that other factors having a spatial pattern which were not taken into account in the analysis, such as genetic or environmental factors, might also be implicated. These results will have to be confirmed in observational studies *in situ*, with objective measures at an individual level.

## Competing interests

The authors declare that they have no competing interests.

## Authors' contributions

MJM conceived of the study, participated in its design and coordination and helped to draft the manuscript. MJM, AP, RB, RR, and GLA performed the statistical analysis and interpretation of data and prepared the draft manuscript. All authors participated in the design of the study and in critical review of the manuscript. All authors read and approved the final manuscript.

## Pre-publication history

The pre-publication history for this paper can be accessed here:

http://www.biomedcentral.com/1471-2458/12/174/prepub
